# Immunostimulatory activity of *Stachys mialhesi* de Noé

**DOI:** 10.1186/1710-1492-9-2

**Published:** 2013-01-11

**Authors:** Assia Benmebarek, Sakina Zerizer, Souheila Laggoune, Zahia Kabouche

**Affiliations:** 1 Laboratoire de Génie microbiologie et application, Equipe: biologie moléculaire et cellulaire. Faculté des sciences, Université de Constantine 1, Constantine, Algeria; 2 Laboratoire d’obtention de substances thérapeutiques (Lost), Université de- Constantine 1, Laboratoire d’Obtention de Substances Thérapeutiques (L.O.S.T), Campus Chaabat Ersas, Constantine, 25000, Algeria

**Keywords:** Immunostimulatory activity, Phagocytic activity, Carbon clearance rate, Reticuloendothelial system, *Stachys mialhesi* de Noé

## Abstract

**Background:**

Immunostimulatory therapy is now being recognized as an alternative to conventional chemotherapy for a variety of disease conditions, involving the impaired immune response of the host. In the present study, the immunostimulatory effect of the butanolic extract obtained from *S. mialhesi* aerial parts, was evaluated *in vivo*.

**Methods:**

The immunostimulant potential of the plant extract on the phagocytic activity was measured by the carbon clearance rate test.

**Results:**

Our research revealed that at different doses (50, 100 and 500 mg/kg), *S. mialhesi* extract increased the phagocytic activity in a dose dependant manner when compared with the control and thus the clearance rate of carbon was faster after the administration of the plant extract.

**Conclusion:**

*S. mialhesi* extract exhibited a dose-dependent immunostimulant effect on the reticuloendothelial system, which could be attributed to the presence of active principles in this butanolic extract.

## Background

Immunostimulators have been known to support T-cell function, activate macrophages, granulocytes, complement and natural killer cells apart from affecting the production of various effectors molecules generated by activated cells (Paraimmunity) [1].

It is expected that non-specific effects offer protection against different pathogens, including bacteria, fungi, and viruses and constitute an alternative to conventional chemotherapy [2].

Immunostimulatory therapy is now being recognized as an alternative to conventional chemotherapy for a variety of disease conditions, involving the impaired immune response of the host [3].

The genus *Stachys* (Lamiaceae) includes about 200 to 300 species in the world [4] and is considered to be one of the largest genera of this family. In Algeria, this genus is represented by 14 species including the endemic species *S. mialhesi* de Noé [5].

Pharmacological studies have confirmed that extract of plants belonging to the genus *Stachys* exert significant antibacterial, anti-inflammatory, antitoxic, anti-nephritic, antihepatitis, anti-anoxia and hypotensive activity, antispasmodic, anti-asthma and anti-rheumatic activities [6] and [7].

In Iran, the aerial parts of *S. inflata* Benth are used to treat infection, asthma, rheumatic and other inflammatory disorders [8]. *S. lavandulifolia* Vahl was used as an anxiolytic and sedative [9].

The study of [10] showed inhibitory effects of *S. obtusicrena* on both cellular and humoral immune responses and suggests that this effect may in part be due to the induction of apoptosis in proliferative lymphocytes.

The stimulation index of all cultures treated with different concentrations of the extract of *S. obtusicrena* was less than 1 [10].

The present investigation was undertaken to evaluate the immunostimulatory effect of the butanolic extract obtained from the aerial parts of *S. mialhesi*, using *in vivo* model.

## Materials and methods

### Plant material

Aerial parts of *S. mialhesi* de Noé were collected in April 2005 at Djebel El-Ouahch Constantine (North Eastern Algerian). The voucher specimen was identified by Professor Gérard De Bélair (University Badji-Mokhtar, Annaba) and was deposited at the Musée botanique de la Ville d’Angers (France) under the reference MBAng2005.10.

### Preparation of the extract

Air-dried and powdered aerial parts (1 kg) of *S. mialhesi* were extracted with 70% MeOH. The residue was suspended in water and extracted successively with petroleum ether, dichloromethane, ethylacetate and n-BuOH.

### Animals

Adult male *Mus Musculus* mice (2.5- 3 month old) from central pharmacy Algeria, weighing (25–36 g) were used for determination of the phagocytic activity. The animals were kept under standard laboratory conditions of humidity, temperature (25± 1°C) and light (12 h day: 12 h night), and allowed free access to food and water.

### Phagocytic activity

The clearance rate of carbon was measured by the method of [11]*.*

Animals were divided into four groups, consisting of 5 mice in GI, GII and GIV but the GIII consisting of 4 mice. Group I (Control) was given 0.9% NaCl (0.5 ml/mouse, i.p), groups II, III and IV were administered by i.p injection with different concentrations of *S. mialhesi* extract (50, 100 and 500 mg/kg) respectively.

After 48 h of i.p injection, the mice were administered with carbon ink suspension at a dose of 0.1 ml/10 g through the tail vein; the mixture consisted of black carbon ink 3 ml, saline 4 ml and 3% gelatin solution 4 ml.

Blood samples were taken from the retro orbital vein by using glass capillaries, at 5 and 15 min. Blood sample drops (14) were mixed with 0.1% sodium carbonate solution (4 ml) for the lysis of erythrocytes and the absorbance measured at 675 nm using a spectrophotometer.

The phagocytic activity is expressed by the phagocytic index K which measures all the reticuloendothelial system function in the contact with the circulating blood. The clearance rate is expressed as the half-life period of the carbon in the blood (t_1/2_, min). These are calculated by means of the following equations:

(1) $$K=\frac{\left({\mathrm{lnOD}}_{1}-{\mathrm{lnOD}}_{2}\right)}{\left(t_{2}-t_{1}\right)},t_{1/2}=0.693/K$$

Where OD_1_ and OD_2_ are the optical densities at times t_1_ and t_2_ respectively.

### Statistical analyses

Results were analyzed for differences between the groups across dietary treatments by one –way ANOVA test and Tukey’s multiple comparison tests (SPSS version 9).

## Results

Effect of *S. mialhesi* extract on the phagocytic activity and the carbon clearance rate is shown in Table 1.

**Table 1:** **Effect of *S. mialhesi *extract on the phagocytic activity and the clearance rate of carbon from the circulating blood of mice**

**Treatment groups**	**Dose**	**Number of mice**	**Phagocytic index K**	**Average phagocytic index K**^***a)***^	**Carbon clearance rate (t**_**1/2**_**,min)**	**Average Carbon clearance rate (t**_**1/2**_**,min)**
Group I Control (Saline)	0.5	5	0.0063	0.032 ^*c)d)*^ ± 0.01	110	41.18 ^*b)*^ ± 18,27
			0.015		46.2	
			0.028		24.75	
			0.054		12.83	
			0.057		12.15	
Group II	50 mg/kg	5	0.013	0.080 ± 0.32^*c)*^	53.30	19.66 ^*b*^ ± 9,07
			0.029		23.89	
			0.072		9.62	
			0.086		8.05	
			0.20		3.46	
Group III	100 mg/kg	4	0.11	0,063 ± 0.015^*c*)^	6.3	
			0.043		16.11	
			0.047		14.74	
			0.053		13.07	
Group IV	500 mg/kg	5	0.085	0.138 ± 0.032 ^*c)d)*^	8.15	5.97 ^*b*^ ± 1,03
			0.097		7.14	
			0.10		6.93	
			0.15		4.62	
			0.26		2.66	

*a)* Each value represents the mean S.E. Difference from the control *b)* p>0.05 and *c)* p=0,05 and *d)* p<0,05.

The present data showed that there is a difference in the means for the phagocytic index (K) between groups (GI, GII, GIII and GIV) P=0,058 and the group IV is significantly different from GI at P= 0,041.

**Figure 1:**
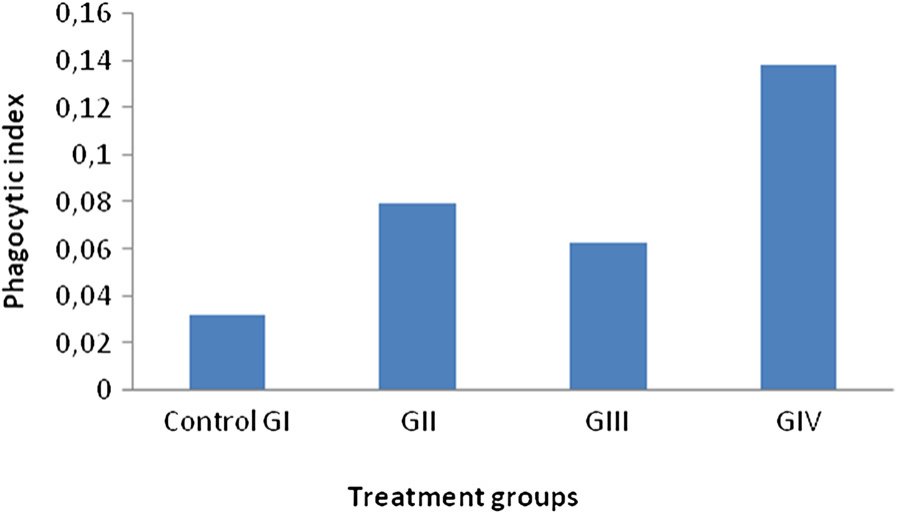
**Effect of *S. mialhesi *extract on phagocytic activity.**

This indicates that *S. mialhesi* extract enhanced the phagocytic activity by stimulating the reticuloendothelial system in a dose dependant manner (Figure 1).

**Figure 2:**
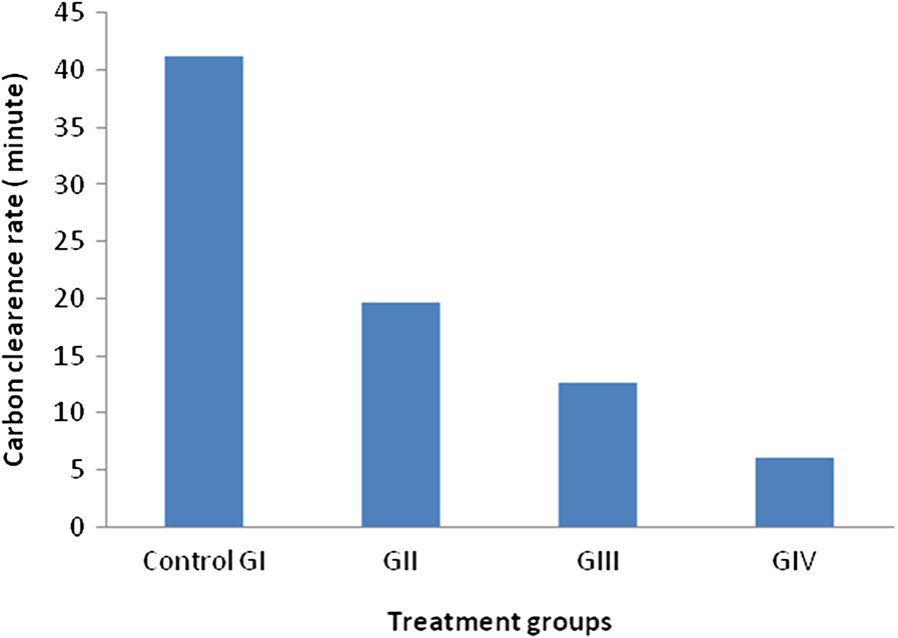
**Effect of *S. mialhesi *extract on the carbon clearance rate.**

As shown in Figure 2, the clearance rate of carbon was faster after 48 h but not significantly between (GI, GII,GIII and GIV) groups P=0,149.

Pearson correlation of phagocytic index (K) and carbon clearance = −0,570 and P= 0,011.

This indicates that the extract shows carbon clearance enhancing activity and affirms that *S. mialhesi* extract enhanced the phagocytic activity.

## Discussion

The reticuloendothelial system is clearing particulate substances, such as bacteria, and altered endogenous materials, such as fibrin aggregates [12]. Phagocytosis is the mechanism by which microorganisms and foreign bodies, dead or injured cells are removed.

Measurement of the activity of the reticuloendothelial system depends upon estimation of the rate of clearance from the blood of foreign materials, such as colloidal carbon [12].

Treatment by *S. mialhesi* extract enhanced the rate of carbon clearance from the blood when compared to the control group. This reflects the enhancement of the phagocytic activity of mononuclear macrophage and nonspecific immunity, which includes opsonisation of the foreign particulate matter with antibodies and complement C3b, leading to a more rapid clearance of foreign particulate matter from the blood [13].

[14] demonstrated that cocoa polyphenols are powerful stimulators of the innate immune system and the first reaction of the adaptive immunity. The immunostimulatory effect was showed following the isolation and culture of purified monocytes, CD4 and CD8 cells in the presence of cocoa polyphenols and after they were confronted with lipopolysaccharides.

Our results revealed that *S. mialhesi* extract appeared to stimulate the phagocytic activity by increasing the clearance rate of carbon by the cells of the reticuloendothelial system. This is due to the fact that it contains natural physiologically active substances such as Terpenoids, Flavanoids and phenolic compounds [15].

## Conclusion

Qualitative phytochemical analysis of the *S. mialhesi* extract revealed that it contains natural physiologically active substances such as Terpenoids, Flavanoids and phenolic compounds [15], and it is already reported that naturally occurring phenolic compounds have immunomodulatory activity [16].

The present study established that *S. mialhesi* extract stimulated non specific immune response of the animals by increasing the phagocytic activity, measured in terms of phagocytic index and this could be attributed to its natural components.

This stimulation indicates the presence of active principles in the *S. mialhesi* extract. The active principles responsible for the stimulatory effect are yet to be identified.

## Abbreviations

Ip: Intraperitoneal; S: Stachys.

## Competing interests

The authors declare that they have no competing interests.

## Authors’ contributions

AB and SZ carried out the studies, acquired data, performed data analysis, drafted and revised the manuscript, and played a major role in all the experimental procedures of this study. SL and ZK carried out the phytochemical part. All authors read and approved the final manuscript.
